# 

**Published:** 1849-07

**Authors:** 


					﻿WHOLE SERIES---VOL. VI, NO. II.
> Vol. II.]
JULY, 1849.
[No. 2.^
THE NORTH-WESTERN
MEDICAL AND SURGICAL
J 0 U R N A L.
=
b ■■
2 ■:
O
S
cd
M
HH )
h :
I °
i* !
1 >
H ;
Q 1
W
in
t-H
iJ
M
«
H
3
O
d
o
ir>
fS
cn
td
W
►
2
2
d
S
M
2
>
a
<5
>
2
Q
M
EDITED BY
JOHN EVANS, M.D.,
PROFESSOR OF OBSTERICS ETC., IN RUSH MEDICAL COLLEGE.
AND
EDWIN Ct. ME1lK, M.D.
CHICAGO AND IND * OLIS:
W. P. DUZAN, Pli NTER.
1849.
ENLARGED SERIES-VOL. IV, NO. II.
				

